# Annotation and curation of the causality information in LncRNADisease

**DOI:** 10.1093/database/baz150

**Published:** 2020-01-15

**Authors:** Kaiwen Jia, Yuanxu Gao, Jiangcheng Shi, Yuan Zhou, Yong Zhou, Qinghua Cui

**Affiliations:** 1 Department of Biomedical Informatics, Department of Physiology and Pathophysiology, Center for Noncoding RNA Medicine, MOE Key Lab of Cardiovascular Sciences, School of Basic Medical Sciences, Peking University, 38 Xueyuan Rd, Beijing, 100191, China; 2 Sanbo Brain Institute, Sanbo Brain Hospital, Capital Medical University, 50 Yikesong Rd, Beijing, 100093, China

## Abstract

Disease causative non-coding RNAs (ncRNAs) are of great importance in understanding a disease, for they directly contribute to the development or progress of a disease. Identifying the causative ncRNAs can provide vital implications for biomedical researches. In this work, we updated the long non-coding RNA disease database (LncRNADisease) with long non-coding RNA (lncRNA) causality information with manual annotations of the causal associations between lncRNAs/circular RNAs (circRNAs) and diseases by reviewing related publications. Of the total 11 568 experimental associations, 2297 out of 10 564 lncRNA-disease associations and 198 out of 1004 circRNA-disease associations were identified to be causal, whereas 635 lncRNAs and 126 circRNAs were identified to be causative for the development or progress of at least one disease. The updated information and functions of the database can offer great help to future researches involving lncRNA/circRNA-disease relationship. The latest LncRNADisease database is available at http://www.rnanut.net/lncrnadisease.

## Introduction

Long non-coding RNAs (lncRNAs) with lengths of more than 200 nucleotides are known to play important roles in contributing to a large variety of diseases, such as cancer and cardiovascular diseases (1–3), and are used as biomarkers in cancer diagnoses (4). As the researches focusing on the relationship between lncRNA and disease accumulated over the past decade, it became necessary to integrate the reported-associations to get a systematic overview of the lncRNA-disease relationships. For this purpose, we launched our first release of the lncRNA disease database (LncRNADisease) (5) in 2012 and developed the LncRNADisease 2 (6) in 2018 with a 40-fold expansion in volume and the integration of associations between circular RNAs (circRNAs) and diseases.

Among all kinds of lncRNA–disease associations, the causal ones have received the most attentions, because they can provide the strongest evidence related to pathogenesis (7). Hence, screening the causal associations is of great importance, and such causality information should be included in the database. However, current LncRNADisease and other similar databases (8,9) do not provide such information. Given this, we updated the LncRNADisease by integrating the causality information of the lncRNA–disease associations. To our knowledge, it is the first lncRNA–disease association database that provides causality information. The updated information and functions may shed substantial light on future studies in this area.

**Figure 1:**
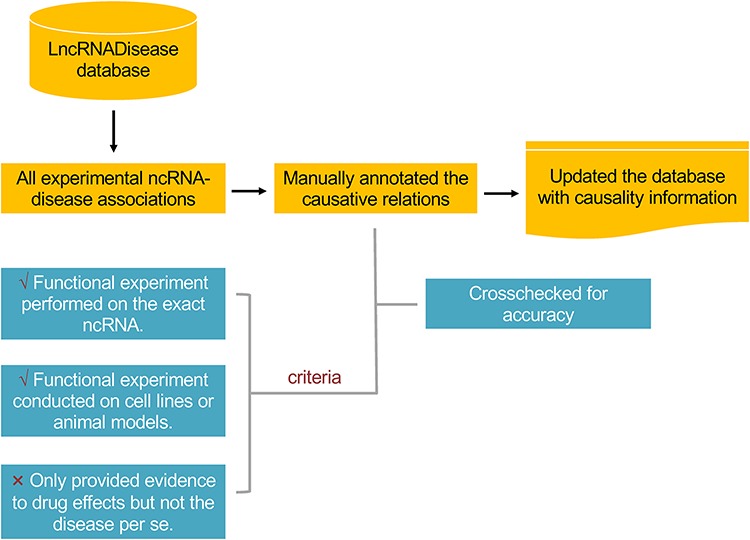
The workflow in annotating the causality information for lncRNA– and circRNA–disease associations.

## Results

**Figure 2:**
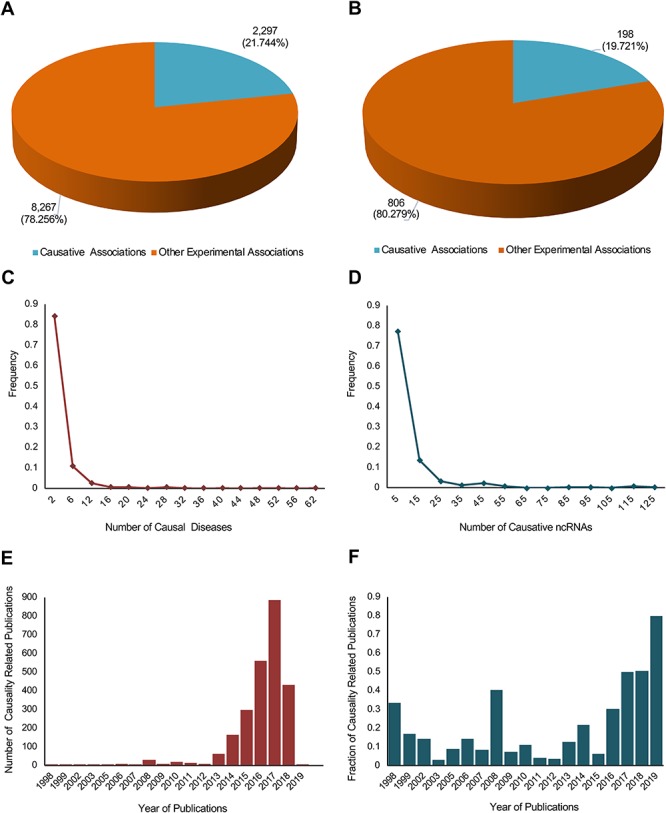
A statistical profile of the causality information of the new update of lncRNADisease. Pie charts show the distribution of causal lncRNA–disease (**A**) and circRNA–disease (**B**) associations. In total, 2297 out of 10 564 lncRNA–disease associations and 198 out of 1004 circRNA–disease associations were identified to be causal. Distribution plots show the number of causal diseases for causative ncRNAs (**C**) and the number of causative ncRNAs for causal diseases (**D**). Bar plots show the number of causality related publications (**E**) and the fraction of causality related publications (**F**).

This update mainly focused on providing the causality information of lncRNA– and circRNA–disease associations. We defined disease causative lncRNAs and circRNAs as those that could directly contribute to the development or progress of a disease. The workflow is shown in Figure **1**. Of the total 11 568 experimental associations, around one fifth of both lncRNA– and circRNA–disease associations were identified to be causal, including 2297 out of 10 564 for lncRNAs and 198 out of 1004 for circRNAs (Figure **2**A and B). In total, 635 lncRNAs and 126 circRNAs were identified to be causative for at least one disease. NcRNAs (lncRNAs and circRNAs) with the greatest number of causal diseases are listed in Table **1**. Diseases with the greatest number of causative ncRNAs are all cancers (Table **2**), which may be due to the complex nosogenesis of cancers. Distribution plots show that most causative ncRNAs have less than two causal diseases, and most causal diseases have less than five causative ncRNAs (Figure **2**C and D). The number of causality related publications and the fraction of these publications have been significantly increasing since 2013 (Figure **2**E and F).

**Table 1:** LncRNAs and circRNAs with the greatest number of causal diseases

lncRNA category	lncRNA symbol	Number of causal diseases
lncRNA	MALAT1	62
	HOTAIR	50
	H19	45
	MEG3	37
	TUG1	30
	GAS5	29
	PVT1	28
	UCA1	26
	NEAT1	25
	CDKN2B-AS1	22
	CCAT1	22
circRNA	hsa_circ_0000284	9
	CDR1-AS	7
	circ-Foxo3	5
	hsa_circ_0001313	4

**Table 2:** Diseases with the greatest number of causative ncRNAs

Disease name	Number of causative ncRNAs
Hepatocellular carcinoma	121
Stomach cancer	113
Colorectal cancer	108
Non–small-cell lung carcinoma	88
Breast cancer	82
Osteosarcoma	55
Lung cancer	53
Prostate cancer	44
Glioma	43

In addition, to provide some implications in molecular mechanism, we categorized the causal associations into several dysfunction patterns (annotated in the last version), including expression, regulation and interaction patterns, which represent different levels in regulation of gene expression when contributing to the development or progress of a disease (Table **3**). Causal associations have more disagreements than other (annotated as not causal) associations in regulation patterns, for 7.08% (76 out of 1073) causal associations have references for both up- and downregulations, and 1.64% (98 out of 5974) for other associations. This result may be due to the research bias for the causative ncRNAs.

**Figure 3:**
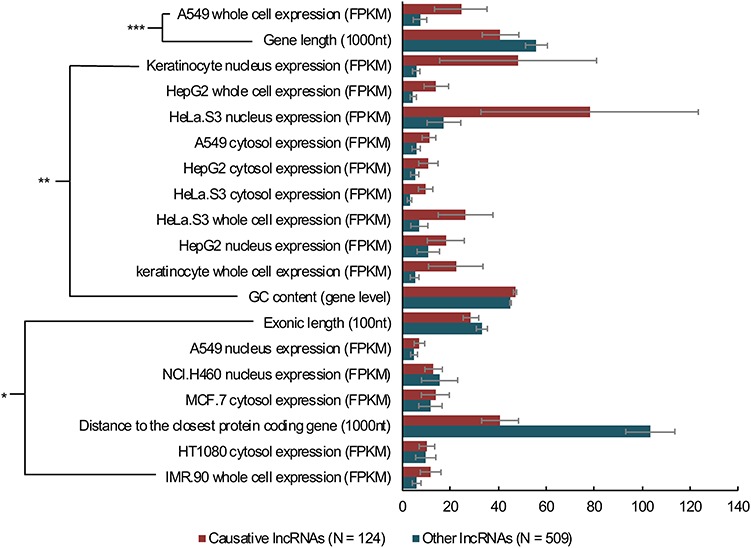
The comparisons between causative and other human lncRNAs. Wilcoxon rank-sum tests were performed on the webserver of LnCompare for comparison analyses of 124 causative human lncRNAs and 509 other (manually annotated as non-causative) human lncRNAs using 109 features. The analysis only performed on lncRNAs with Ensembl IDs. *P < 0.05, **P < 0.01, ***P < 0.001, error bars show the SEM.

Moreover, we compared causative human lncRNAs with non-causative ones in the database with 109 features using an online tool LnCompare (10), and the significant results are shown in Figure **3**. The causative lncRNAs have higher expression level in different cell types, higher GC content, shorter distance to the closest protein coding gene and shorter gene and exon length.

## Materials and methods

### Data collection

We downloaded the MySQL database from the latest version of LncRNADisease to ensure all data was collected in the original database. Experimental associations with references (11 568 in total) were extracted from the corresponding table of the database.

**Table 3:** Statistical summary for the dysfunction patterns of the causal associations

Dysfunction pattern	Causal association counting
Expression [high/over expression]	311
Expression [low expression]	61
Regulation [upregulated]	984
Regulation [downregulated]	309
Interaction/regulation [microRNA]	525
Interaction/regulation [protein]	206
Interaction/regulation [mRNA/lncRNA]	5

The dysfunction patterns were extracted and counted from the annotations in the database.

### Annotation for the causal associations

We manually annotated the causal associations by reviewing the abstracts of related publications. Causal associations were identified by the corresponding following criteria: (i) Functional experiments like gain-of-function and/or loss-of-function experiments must be performed on the exact lncRNAs or circRNAs and (ii) functional experiments must be conducted on cell lines and/or animal models of human diseases. Associations were excluded if the research only acknowledged the relationships between lncRNA or circRNA and drug effects but not the disease per se. To ensure the accuracy of the curation, the annotated causality information had been crosschecked by different researchers. In total, 2531 associations were annotated to be causal at first, whereas 70 were removed and another 34 were added after crosscheck. As the criteria were quite clear, the disagreements in annotation were mainly due to wrong judgments led by manual work.

### Implementation

We updated the MySQL database of LncRNADisease 2.0 on the website with causality information. The web interface for browsing and searching was implemented by PHP and JavaScript programs. Apache Tomcat web server was used for the http server.

### Database usage

Users can browse all the lncRNA– and circRNA–disease causal associations using the ‘Causality’ filter on the BROWSE page, with ‘Yes’ option means at least one reference in the associations is annotated to be causal, ‘Unknown’ option means no references in the associations are annotated to be causal, and ‘Not Available’ option for predicted associations or experimental associations with references no longer available in the PubMed.

In addition, two new sections ‘ncRNA Association Statistics’ and ‘Disease Association Statistics’ are available in the Entry Detail page (users can enter the Entry Detail page from Search Results page or BROWSE page). In these two sections, total associated disease/non-coding RNA (ncRNA) number, causal disease/ncRNA number, a network of each ncRNA/disease and its associated diseases/ncRNAs are shown. More information for each associated disease/ncRNA, such as species, IDs, definitions and its causality information, are also provided for users to browse and download for further analysis. The causality information (Yes/No) is also added in each reference of an association on the Entry Detail page. ‘Yes’ or ‘No’ means that in which reference this association is annotated to be causal or not causal.

Users can also download all the causal associations on the DOWNLOAD page. The STATISTICS, SUBMIT and HELP pages were updated as well, with changes related to causality information.

### Perspectives and concluding remarks

Disease causative lncRNAs and circRNAs directly contribute to the development or progress of a disease relative to others that are passively altered during a disease process. The identification of the disease causative ncRNAs is of great importance for understanding how they contribute to diseases (7,11). The causality information we updated provides a valuable source for future researches, such as defining the research potential of the disease-related ncRNAs, screening the ncRNA drug targets or predicting the causal ncRNA-disease associations.

With the identified causative lncRNAs and circRNAs, some function implications can offer help to see whether causative and other ncRNAs function differently in a disease. However, currently, there is no tool that can directly perform the functional enrichment analysis for lncRNAs/circRNAs. Therefore, we only provide basic information for all associated lncRNAs and circRNAs for a given disease in this version. We may introduce the function enrichment result to the database when lncRNA enrichment analysis tools are available.

## Authors’ contributions

Q.C. conceived the project. K.J. curated the causality of each items in reference table of the database. Y.G checked K.J.’s annotation results and updated the database in the web server. K.J. prepared the figures and wrote the manuscript. Q.C. thoroughly revised the manuscript. J.S., Y.Z. and Y.Z. provided valuable suggestions. All authors discussed the results and contributed to the final manuscript.
